# Perforation of the neovagina in a male-to-female transsexual: a case report

**DOI:** 10.1186/1752-1947-9-24

**Published:** 2015-01-23

**Authors:** Yuto Shimamura, Aoi Fujikawa, Keisuke Kubota, Naoki Ishii, Yoshiyuki Fujita, Keiichiro Ohta

**Affiliations:** 1 Division of Gastroenterology, St. Luke’s International Hospital, 9-1 Akashi-cho, Chuo-ku, Tokyo, 104-8560 Japan; 2 Division of General Surgery, St. Luke’s International Hospital, 9-1 Akashi-cho, Chuo-ku, Tokyo, 104-8560 Japan

**Keywords:** Neovagina, Peritonitis, Sigmoid vaginoplasty, Transsexual

## Abstract

**Introduction:**

There are several techniques for creation of a neovagina in male-to-female reassignment surgery. Although vaginoplasty with the sigmoid colon is not a common procedure, it is becoming more common. Perforation of the recto-sigmoid neovagina after sex reassignment surgery is very rare. We hereby report a case of perforation of the neovagina that presented as acute peritonitis, with a massive abscess in the intra-abdominal cavity.

**Case presentation:**

This case report describes a 33-year-old Asian woman presenting with mild persistent abdominal pain, nausea, and vomiting who had undergone male-to-female sex reassignment surgery four years prior. Physical examination revealed mild abdominal pain without rebound tenderness. An abdominal computed tomography scan showed a massive abscess that occupied a significant portion of the intra-abdominal cavity. Perforation of the neovagina was confirmed by exploratory laparotomy and surgical drainage with primary closure was performed without any complications.

**Conclusion:**

This is a rare case involving perforation of the neovagina that was successfully treated with surgical intervention. This case emphasizes the importance of taking a detailed medical history and to make physicians and patients aware that bowel vaginoplasty can result in a weak vagina.

## Introduction

Vaginal reconstruction is indicated in several conditions including vaginal agenesis, gender dysphoria in biological males, and genital trauma. There are several techniques for construction of the neovagina in male-to-female reassignment surgery. The penile-scrotal skin flap technique is considered the standard in vaginoplasty, however, use of the sigmoid colon for reconstruction is becoming more common because of good sexual and psychosocial outcomes [1–6]. Although complications have been reported, including neovaginal prolapse, introital stenosis, and unsatisfied female sexual function [7, 8], perforation of the neovagina is very rare [9, 10]. We hereby report a case of perforation of the neovagina that presented as acute peritonitis with a massive abscess in the intra-abdominal cavity. The perforation of the vaginal vault was confirmed intraoperatively, and surgical drainage was performed successfully without any complications.

## Case presentation

A 33-year-old Japanese female who underwent male-to-female sex reassignment surgery four years prior presented to our clinic with persistent abdominal pain that had gradually worsened over seven days. She also complained of nausea and vomiting, but otherwise claimed to be healthy. She had visited her primary care physician five days earlier, but no definite diagnosis was made.

**Figure 1:**
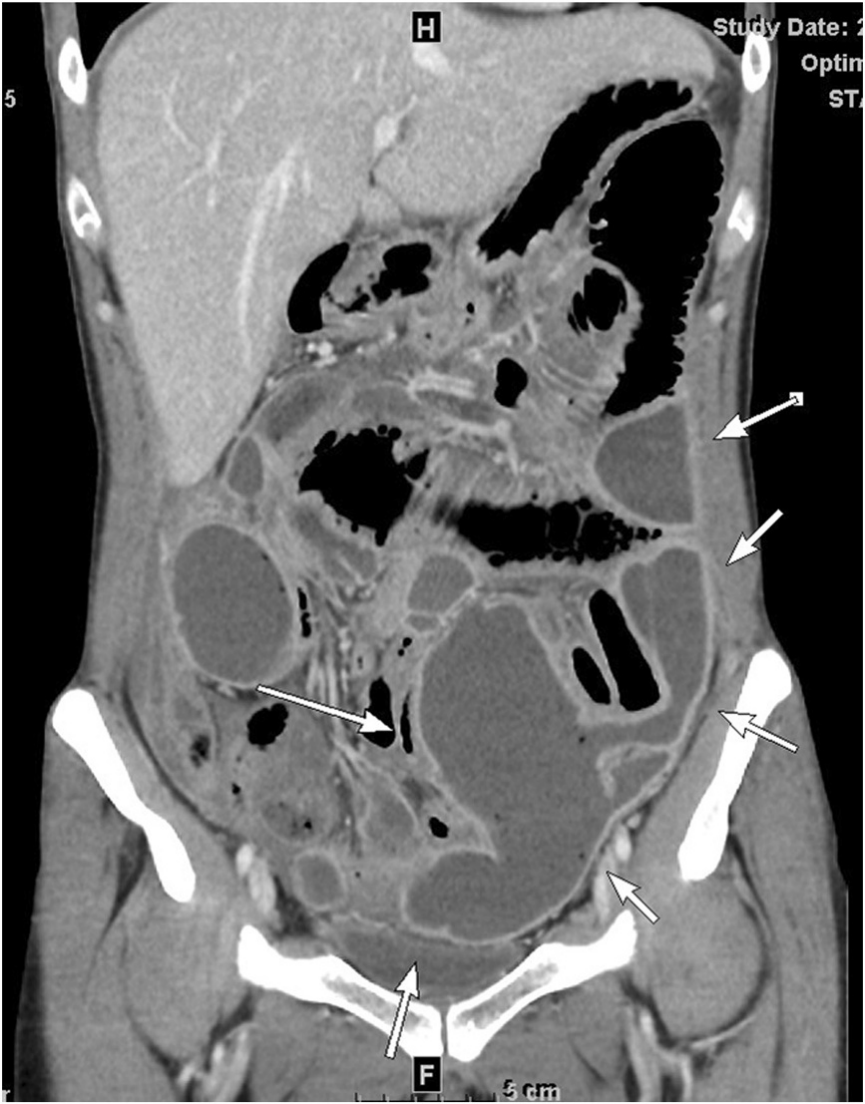
**Abdominal computed tomography (CT) scan.** An abdominal CT scan showed a massive abscess occupying a significant portion of the intra-abdominal cavity. A well-circumscribed abscess fluid collection with enhanced walls are seen as pointed by the white arrows.

Upon presentation, the patient was alert and vital signs were unremarkable except for a fever of 38°C. Physical examination revealed mild abdominal tenderness without rebound tenderness. There were no other remarkable findings. A blood test revealed an increased white blood cell count (12,600/μL) and C-reactive protein level (26.64mg/dL), and liver and kidney function tests were normal. An abdominal X-ray showed signs of bowel obstruction with bowel distention and multiple gas-fluid levels. An abdominal computed tomography scan revealed partial bowel obstruction and a massive abscess, which occupied a significant portion of the intra-abdominal cavity (Figure 1), but no free air was apparent. Further history taking revealed that the sigmoid colon was used to construct the neovagina. The patient had no major complications other than mild stenosis of the neovagina and was advised by her primary surgeon to undergo vaginal dilation via daily insertion of plastic stents (2–4×20cm) 10cm into her neovagina. The patient stated that she had vaginal intercourse and vaginal dilation a few days prior to the onset of her symptoms.

**Figure 2:**
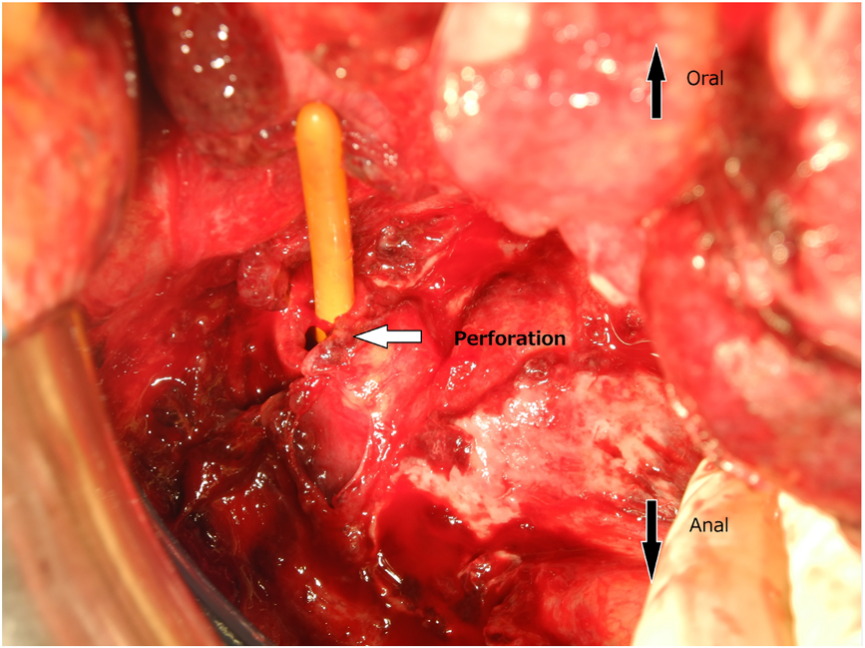
**Exploratory laparotomy.** Perforation of the neovagina was confirmed and primary closure was performed.

Although free air was not observed in radiographic images, bowel perforation was suspected. However, considering the discrepancy between the mild clinical manifestations and the abdominal imaging, we suspected a perforation of the neovagina rather than of the intestinal tract. Intravenous antibiotics were immediately administered, and surgical intraperitoneal drainage was conducted five hours after initial presentation to our hospital. During the procedure, 10L of saline was used, and the perforation was confirmed at the top of the neovagina (Figure 2). Primary closure using slow absorbable 3-0 sutures was performed. The exact volume of the neovagina was not measured, but it was at least 10cm in length and there was no obvious introital stenosis at the time of operation. No perforation of the intestinal tract was found during the operation, and the relationship between the perforation and vaginal dilation could not be determined. Although we observed adhesion in the small intestine over a broad range, we were able to completely drain the intra-abdominal abscess and the surgical intervention itself was uneventful. There were no complications related to the surgery, though eight weeks of intravenous antibiotics for multidrug resistant bacteria were continued in case of a residual abscess.

## Discussion

Perforation of the neovagina after sex reassignment surgery is very rare. In 2001, Liguori *et al.*[9] reported a case of acute peritonitis due to introital stenosis that subsequently caused perforation of the neovagina constructed from bowel. A second case was reported by Amirian *et al.*[10] in 2011, involving a patient that was conservatively treated with antibiotics. In the present case, a neovaginal perforation was the suspected cause of the intra-abdominal abscess because of the discrepancy between the symptomology and the radiologic findings. Although conservative treatment with antibiotics was considered in our case, we performed surgical intraperitoneal drainage based on the clinical judgment that the abscess may be refractory to antibiotic treatment. As a result, the patient recovered quickly without any perioperative complications.

Spontaneous rupture of the neovagina with introitus occlusion has been reported, however, perforation without introital stenosis has not been described. In contrast to previous literature that emphasizes the importance of regular sexual intercourse and vaginal dilation to avoid complete introital stenosis [5], we speculate that these practices may have caused the perforation, allowing colonic bacteria to leak into the abdominal cavity, and resulting in the formation of an abscess.

## Conclusion

We report a case involving perforation of the neovagina that was successfully treated with surgical intervention. Rupture of the neovagina is a very rare origin of an acute abdominal syndrome, and this case emphasizes the importance of taking a detailed medical history and making physicians and patients aware that bowel vaginoplasty can result in a weak vagina.

## Consent

Written informed consent was obtained from the patient for publication of this case report and accompanying images. A copy of the written consent is available for review by the Editor-in-Chief of this journal.

## References

[1] Wroblevski P, Gustafsson J, Selvaggi G: **Sex reassignment surgery for transsexuals.** *Curr Opin Endocrinol Diabetes Obes* 2013, **20:**570–574. 10.1097/01.med.0000436190.80104.56 10.1097/01.med.0000436190.80104.56 24468760

[2] Selveggi G, Bellringer J: **Gender reassignment surgery: an overview.** *Nat Rev Urol* 2011, **8:**274–282. 10.1038/nrurol.2011.46 10.1038/nrurol.2011.46 21487386

[3] Djordjevic ML, Stanojevic DS, Bizic MR: **Rectosigmoid vaginoplasty: clinical experience and outcomes in 86 cases.** *J Sex Med* 2011, **8:**3487–3494. 10.1111/j.1743-6109.2011.02494.x 10.1111/j.1743-6109.2011.02494.x 21995738

[4] Labus LD, Djordjevic ML, Stanojevic DS, Bizic MR, Stojanovic BZ, Cavic TM: **Rectosigmoid vaginoplasty in patients with vaginal agenesis: sexual and psychosocial outcomes.** *Sex Health* 2011, **3:**427–430. 10.1071/SH10105 21851786

[5] Kwun S, Hoon J, Cheol K, Min J, Tae J, Chan M: **Long-term results in patients after rectosigmoid vaginoplasty.** *Plast Reconstr Surg* 2003, **112:**143–151. 10.1097/01.PRS.0000066169.78208.D4 10.1097/01.PRS.0000066169.78208.D4 12832887

[6] Kapoor R, Sharma DK, Singh KJ, Suri A, Singh P, Chaudhary H, Dubey D, Mandhani A: **Sigmoid vaginoplasty: long-term results.** *Urology* 2006, **67:**1212–1215. 10.1016/j.urology.2005.12.037 10.1016/j.urology.2005.12.037 16765181

[7] Nowier A, Esmat M, Hamza RT: **Surgical and functional outcomes of sigmoid vaginoplasty among patients with variants of disorders of sex development.** *Int Braz J Urol* 2012, **38:**380–386. 10.1590/S1677-55382012000300011 10.1590/s1677-55382012000300011 22765864

[8] Rawat J, Ahmed I, Pandey A, Khan TR, Singh S, Wakhlu A, Kureel SN: **Vaginal agenesis; experience with sigmoid colon neovaginoplasty.** *J Indian Assoc Pediatr Surg* 2010, **15:**19–22. 10.4103/0971-9261.69136 10.4103/0971-9261.69136 PMC2998662 21180499

[9] Liguori G, Trombetta C, Buttazzi L, Belgrano E: **Acute peritonitis due to introital stenosis and perforation of a bowel neovagina in a transsexual.** *Obstet Gynecol* 2001, **97:**828–829. 10.1016/S0029-7844(00)01154-6 10.1016/s0029-7844(00)01154-6 11336769

[10] Amirian I, Gogenur I, Rosenberg J: **Conservatively treated perforation of the neovagina in a male to female transsexual patient.** *BMJ Case Rep* 2011. doi:10.1136/bcr.08.2010.3241 10.1136/bcr.08.2010.3241 PMC3062371 22714607

